# Conduction Disorders After Transcatheter Aortic Valve Implantation: Evolution Over Time and Association With Long-Term Outcomes

**DOI:** 10.1016/j.shj.2025.100428

**Published:** 2025-02-17

**Authors:** Aileen Paula Chua, Rinchyenkhand Myagmardorj, Takeru Nabeta, Jurrien H. Kuneman, Frank van der Kley, Jeroen J. Bax, Nina Ajmone Marsan

**Affiliations:** a Department of Cardiology, Heart Lung Center, Leiden University Medical Center, Leiden, The Netherlands; b Department of Cardiology, Turku Heart Center, University of Turku and Turku University Hospital, Turku, Finland

**Keywords:** Conduction disorders post-TAVI, Left bundle branch block, Long-term outcomes, Permanent pacemaker

## Abstract

**Background:**

Expanding indications for transcatheter aortic valve implantation (TAVI) highlighted the importance of complications such as new left bundle branch block (LBBB) or permanent pacemaker (PPM) implantation. However, studies on the long-term outcomes of these conduction abnormalities (CA) are limited. This study aims to examine the progression of CA within the first year after TAVI and their long-term prognostic value.

**Methods:**

TAVI patients were divided into 1) PPM implantation within the first year, 2) post-TAVI LBBB persisting until 1 year (permanent LBBB), and 3) no-CA. Endpoint was all-cause mortality after 1 year.

**Results:**

Among 794 patients initially included, 30% developed new LBBB, which persisted in 17% until discharge; 12% received a PPM during the hospitalization. One-year follow-up was available in 502 patients: 11% were classified as permanent LBBB (n = 56), 18% as PPM (n = 89), and the rest as no-CA (n = 357). Baseline characteristics were comparable, except for valve type, with self-expanding more common among the PPM group. At 1-year follow-up, lower left ventricular ejection fraction and global longitudinal strain were observed in the PPM and permanent LBBB groups compared to the no-CA group (55% ± 9% and 15% ± ​4% vs. 54% ± 11% and 15% ± 4% vs. 58% ± 9% and 17% ± ​4%, respectively, *p* ​< 0.001). At long-term follow-up (median: 4 [interquartile range: 3-6] years), higher mortality was observed in the PPM group (ꭓ^2^ = 10.168, *p* = 0.006). In addition, PPM implantation (hazard ratio: 1.654, *p* = 0.011) and global longitudinal strain at 1 year (hazard ratio: 0.950, *p* = 0.027), as well as pre-TAVI EuroSCORE II and New York Heart Association III-IV at 1 year, were independently associated with the outcome.

**Conclusions:**

Post-TAVI CAs are dynamic within the first year. Patients who needed PPM implantation did not show significant improvement in left ventricular function after TAVI and had higher long-term mortality.

## Highlights

• Conduction abnormalities (CA) are dynamic during the first year post-transcatheter aortic valve implantation (TAVI).
• Patients with pacemakers after TAVI show no improvement in left ventricular function at 1 year.
• Patients with pacemakers after TAVI have an increased risk for long-term mortality.
• Global longitudinal strain is useful to monitor post-TAVI patients, especially those who develop CA.

## Graphical abstract

The upper diagram depicts the progression of CA immediately post-TAVI, at discharge, and at 1-year follow-up, and how the groups used in the study were determined. The lower panel shows the change in LVEF and GLS for each CA group from baseline to 1 year, with significant improvement seen only in the no-CA group. Kaplan-Meier analysis starting at 1 year shows a significant difference in outcome with the PPM group.Abbreviations: CA, conduction abnormality; EF, ejection fraction; GLS, global longitudinal strain; LBBB, left bundle branch block; LV, left ventricle; PPM, permanent pacemaker; TAVI, transcatheter aortic valve implantation.

**Figure:**
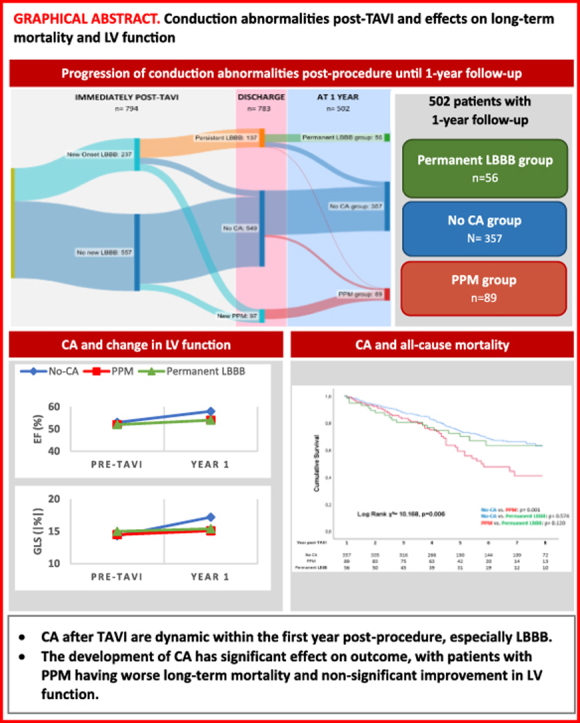

## Introduction

Transcatheter aortic valve implantation (TAVI) is an effective treatment option for patients with severe aortic stenosis (AS), not only for high- and intermediate-risk patients but also for those with lower surgical risk.^1^ Optimization of procedural aspects and valve prosthesis design has greatly improved patient outcomes, especially in the first year after TAVI implantation.^2^^,^^3^ Now, attention is given to the complications that affect long-term outcomes, especially in low-risk patients with longer life expectancies. Conduction abnormalities (CA) are still relatively common after TAVI, including high-grade atrioventricular block (HAVB), left bundle branch block (LBBB), and the need for a permanent pacemaker (PPM).

Numerous studies have evaluated the factors associated with the development of CA and their impact on outcomes after TAVI, but results are inconsistent, and follow-up duration is limited to the first 1 to 2 ​years.^4^ In addition, most studies investigated the occurrence of CA immediately post-TAVI without considering the progression or persistence of electrocardiographic changes during follow-up. Therefore, the current study aims to 1) examine the progression of significant CA within the first year post-TAVI in a large patient cohort; 2) investigate the impact of PPM implantation and permanent LBBB on outcomes, starting from the first year after TAVI; and 3) identify additional factors at follow-up that may be associated with long-term mortality. Since these patients are often referred back to their primary care physician at 1 year after TAVI, understanding the clinical relevance of these complications may help guide risk stratification and monitoring of these patients for the subsequent years.

## Materials and Methods

### Study Population

Patients with severe AS who underwent TAVI between 2007 and 2019 at the Leiden University Medical Center (the Netherlands) were included in the study. Patients with cardiac implantable electronic devices (either PPM or implantable cardiac defibrillator) and preexisting LBBB were excluded. Patients were assessed before the procedure, after the procedure (immediately after and before discharge), and at 1-year follow-up. Long-term analysis was performed only on patients with complete data at 1-year follow-up. These patients were then divided into 3 groups: 1) those who underwent PPM implantation within the first year (PPM group), 2) those who developed LBBB during the post-TAVI hospitalization and persisted until 1-year follow-up (permanent LBBB group), and 3) those without new LBBB or PPM (no-CA group).

The decision to perform TAVI was taken by a multidisciplinary heart team. Clinical data, including demographics, cardiovascular risk factors, medications, and procedural information, were retrospectively collected from the departmental Cardiology Information system (EPD-Vision; Leiden University Medical Center, the Netherlands). Indications for PPM implantation were in accordance with guidelines.^5^ The institutional review board of the Leiden University Medical Center approved the retrospective analysis of the clinically acquired data and waived the need for written informed consent.

### Electrocardiographic Variables

Twelve-lead electrocardiograms (ECGs) were collected at baseline, post-TAVI (immediately after TAVI until discharge), and at 1-year follow-up. ECG calibration was set at 0.1 mV/mm and the paper speed at 25 mm/s. Heart rate and rhythm, PR interval, and QRS duration were recorded. New CA, specifically atrioventricular blocks, LBBB, right bundle branch block, and nonspecific intraventricular conduction defects (IVCDs), were analyzed. The criteria defining conduction disturbances were adopted from the third Valve Academic Research Consortium and the American Heart Association/American College of Cardiology/Heart Rhythm Society recommendations.^6^^,^^7^

### Echocardiographic Variables

Transthoracic echocardiography was performed both at pre-procedure and at 1-year follow-up using commercially available ultrasound systems (Vivid7, VividE9 and E95; GE Healthcare, Horten, Norway) equipped with 3.5 MHz or M5S-D transducers. Parasternal, apical, subcostal, and suprasternal views were obtained according to current recommendations.^8^ Data were digitally stored in cine-loop format for offline analysis using commercially available software (EchoPac 204; GE Medical Systems, Horten, Norway) and were retrospectively analyzed.

From the apical three- or five-chamber views, continuous wave Doppler recordings were measured to estimate peak aortic jet velocity, and the mean transvalvular pressure gradient was calculated using the Bernoulli equation. Aortic valve area was derived from the left ventricular (LV) outflow tract diameter and the velocity-time integrals of the aortic valve (AV) and outflow tract. LV dimensions were measured from the parasternal long-axis view and used to calculate LV mass index (LVMI). The apical four- and two-chamber views provided LV end-systolic and end-diastolic volumes, and ejection fraction (EF) was derived using biplane Simpson’s method. Right ventricular (RV) dysfunction was defined as tricuspid annular plane systolic excursion ​<17 mm. The presence of significant regurgitation of the aortic, mitral, and/or tricuspid valves was assessed by a multiparametric approach based on current guidelines.^8^^,^^9^

LV strain was measured using speckle-tracking imaging (EchoPac204; GE, Horten, Norway). The analysis was performed from the apical two-, three-, and four-chamber views with a frame rate >40 frames/sec. The region of interest was determined automatically but manually adjusted when necessary. Global longitudinal strain (GLS) was calculated from the average peak strain of the 17 LV segments and reported as absolute (i.e., positive) values.

### Clinical Endpoint

The study endpoint was all-cause mortality starting from the 1-year follow-up after TAVI. Data were obtained through the departmental Cardiology Information System, which is linked to the governmental death registry database.

### Statistical Analysis

Continuous variables are expressed as means ​± ​SDs or as medians and interquartile ranges and were compared between the 3 groups using Analysis of Variance (ANOVA) or the Kruskal-Wallis test, as appropriate. Categorical variables are expressed as numbers and percentages and were compared using the chi-square test or Fisher exact test. The Bonferroni method was used to correct for multiplicity. For the comparison of echocardiographic parameters between baseline and follow-up, paired sample t-test for 2 related samples was used.

The survival rate was estimated using Kaplan-Meier analysis and the log-rank test. Analysis was started 12 months after TAVI until the endpoint was reached. To investigate the association between clinical, electrocardiographic, and echocardiographic parameters with all-cause mortality, univariable and multivariable Cox proportional hazards regression analyses were performed. Variables at 1-year follow-up, which were statistically significant on univariable analysis, were included in the multivariable regression. Only EuroSCORE II was included from the baseline characteristics, and the clinical variables comprising the score were excluded in the model to avoid collinearity. ECG parameters after TAVI were likewise not included since these are inherent to the definition of the subgroups. From the variables at the 1-year follow-up, New York Heart Association (NYHA) functional class was selected as a measure of clinical status, while among the echocardiographic parameters, GLS was selected as a measure of LV function, and LVMI as a measure of size, since LV hypertrophy in patients undergoing TAVI has been associated with mortality.^10^ Hazard ratios and 95% CIs were calculated.

All analyses were performed using SPSS for Windows, version 25 (SPSS, Armonk, NY). A *p*-value <0.05 was considered statistically significant.

## Results

### Progression of Conduction Abnormalities After TAVI

**Figure 1:**
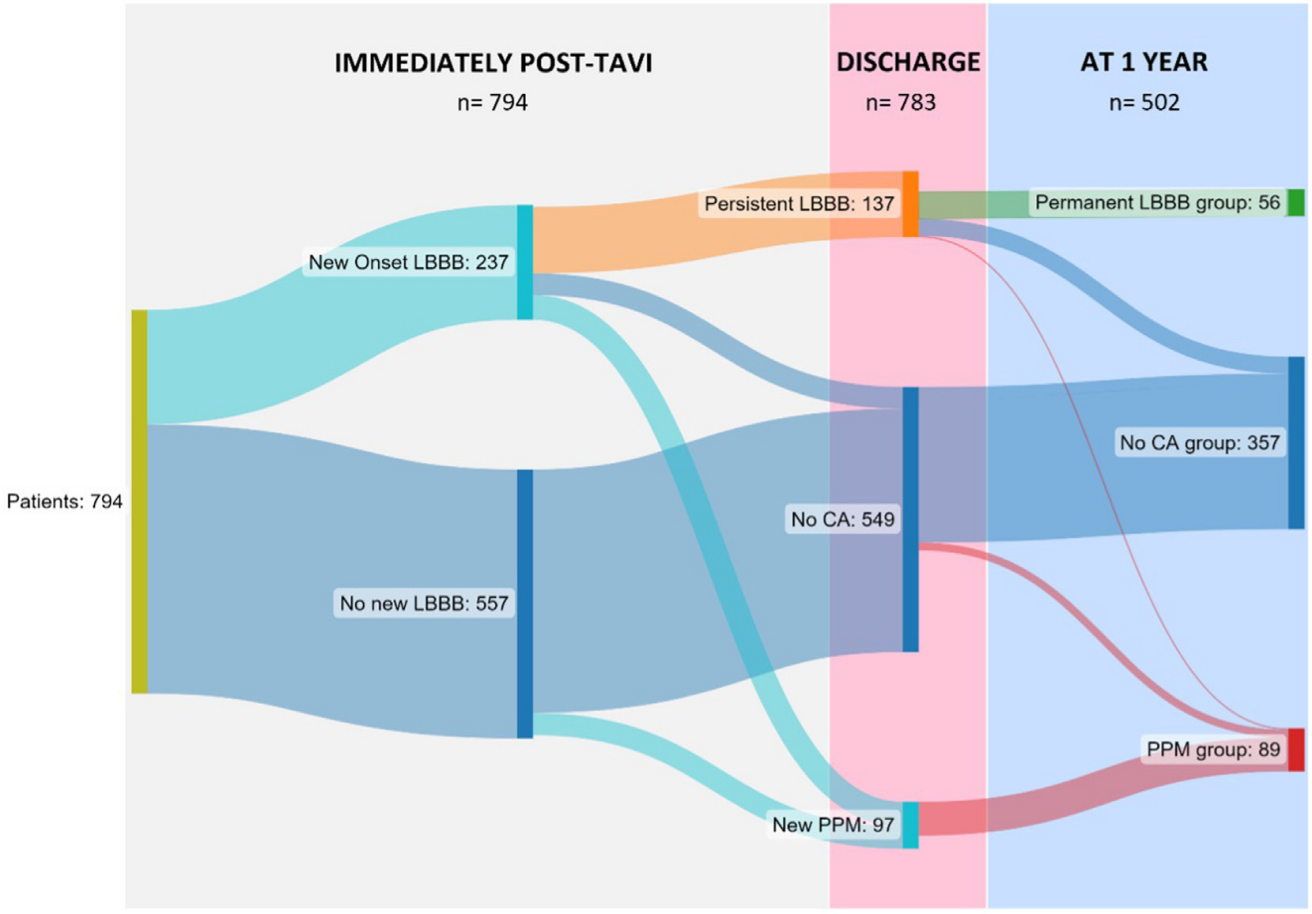
**Progression of CA over time**. This Sankey diagram illustrates the progression of CA assessed in this study at 3 time points: immediately postprocedure, at discharge, and at 1-year follow-up. (*Image made in Sankeymatic*.*com*). Abbreviations: CA, conduction abnormality; LBBB, left bundle branch block; PPM, permanent pacemaker; TAVI, transcatheter aortic valve implantation.

The progression of CA according to the different time points is illustrated in Figure 1.

#### CA Immediately Post-TAVI and at Discharge

An initial group of 794 patients who underwent TAVI and fulfilled the inclusion criteria abovementioned were examined for the preliminary analysis. From these patients, 30% (n = 237) presented with new-onset LBBB immediately post-TAVI, but only 17% still showed LBBB at discharge (n = 137, “persistent LBBB”; in 100 patients, LBBB resolved), while 6% (n = 51) underwent PPM implantation during hospitalization. Of the 70% (n = 557) of patients who did not develop LBBB after TAVI, 8% (n = 46) needed a PPM during the hospital stay, while the remaining patients (n = 549, 62%) did not have either LBBB or PPM at discharge.

#### CA at 1-Year Follow-Up

At 1 year, 205 patients did not have follow-up, and 87 patients died. Therefore, 502 patients who had follow-up ECG 1 year after TAVI were included in the long-term analysis.

Of the patients with LBBB at discharge (94 patients), only 56 still presented LBBB at 1-year follow-up, comprising the permanent LBBB group (since no patients developed new LBBB at 1 year). In 35 patients, LBBB resolved, and they were considered as no-CA, while 3 needed a PPM implantation and were included in the PPM group.

From the 549 patients who did not develop CA at discharge, 16 patients needed a PPM within the first year post-TAVI and were included in the PPM group.

**Figure 2:**
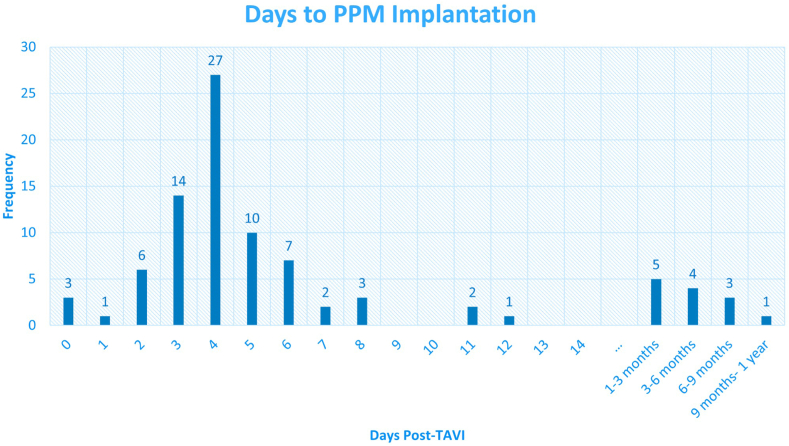
**Timing of PPM implantation from the day of TAVI**. This histogram displays the frequency of time to PPM implantation, with a median time of 4 days after TAVI. Abbreviations: PPM, permanent pacemaker; TAVI, transcatheter aortic valve implantation.

Therefore, when examining the patients at the 1-year follow-up, the PPM group consists of 89 patients (18%) who received a pacemaker within the first year following TAVI. The median time of PPM implantation was 4 days after TAVI, with an interquartile range of 3 to 6 days (Figure 2). Although less common, 21% of patients (n = 19) received the PPM after the first week.

Regarding the type of PPM, dual chamber pacing was most common (54%), followed by single-chamber pacing (32%). In 10% of patients, an implantable cardiac defibrillator or cardiac resynchronization therapy was implanted. Indications for the PPM were high-grade AV block in 73% and sinoatrial node disease in 17%.

Percentage of pacing was assessed at baseline and at follow-up. At implantation, 54% of patients were paced >40% of the time, 16% paced 1% to 40%, while 19% had <1% pacing. At the 1-year follow-up, pacing frequency was slightly reduced, with 45% paced >40% of the time, 29% paced 1% to 40% of the time, and 17% had <1% pacing.

### Long-Term Analysis

#### Baseline Patient Population Characteristics

Since outcomes were explored from 1-year after TAVI, long-term analysis only included the 502 patients with follow-up ECG. Compared to the patients who were excluded (n = ​292; Supplementary Table 1), the study population had higher median EuroSCORE II and more frequent history of coronary artery disease and dyslipidemia. In terms of echocardiographic parameters, end-diastolic and end-systolic volumes were lower, but EF was not significantly different from those who were excluded. GLS was higher in the patients with follow-up.

The study population for long-term analysis was divided into the PPM group (n = 89), permanent LBBB group (n = 56), and no-CA group (n = 357). When comparing the groups (Table 1), preexisting comorbidities were for the most part comparable, except for the presence of peripheral artery disease, which was less frequent in the PPM group relative to the no-CA group. As for the procedural characteristics, the percentage of patients who underwent TAVI using the transfemoral or transapical/subclavian approach was similar. However, valve type significantly differed, with self-expanding valves more frequent among the PPM group compared to the no-CA group (40 vs. 15%, *p* < ​0.001).

**Table 1:** Baseline (pre-TAVI) clinical and echocardiographic characteristics according to the subgroups of CA

Variables	No-CA (n ​= ​357)	PPM (n ​= ​89)	Permanent LBBB (n ​= ​56)	*p*-value
Age (years)	80 ​± ​7	80 ​± ​7	79 ​± ​6	0.724
Sex, male *n* (%)	185 (52)	55 (62)	25 (45)	0.104
BMI (kg/m^2^)	26.5 ​± ​4.1	26.3 ​± ​4.2	28.3 ​± ​6.1∗^,^†	**0**.**010**
EuroSCORE II (%)	2.95 (1.94-5.06)	3.07 (1.86-4.73)	3.45 (2.08-5.14)	0.535
NYHA, *n* (%)				
I-II	145 (41)	39 (44)	19 (34)	0.493
III-IV	212 (59)	50 (56)	37 (66)	
Comorbidities				
Hypertension, *n* (%)	266 (75)	68 (76)	47 (84)	0.307
Dyslipidemia, *n* (%)	231 (65)	58 (65)	39 (70)	0.770
Diabetes mellitus, *n* (%)	94 (26)	21 (24)	21 (38)	0.155
Coronary artery disease, *n* (%)	207 (58)	57 (64)	32 (57)	0.558
Previous MI, *n* (%)	71 (20)	19 (21)	8 (14)	0.550
Stroke, *n* (%)	64 (23)	10 (16)	12 (26)	0.388
PAD, *n* (%)	110 (31)	15 (17)∗	19 (34)	**0**.**022**
Atrial fibrillation, *n* (%)	91 (26)	26 (29)	15 (27)	0.772
Previous valve procedures, *n* (%)	22 (6)	4 (5)	4 (7)	0.777
Smoking, *n* (%)	80 (22)	13 (15)	12 (21)	0.268
COPD, *n* (%)	56 (16)	15 (17)	14 (25)	0.225
eGFR (mL/min/1.73 m^2^)	65 ​± ​22	64 ​± ​20	63 ​± ​22	0.925
Procedural factors				
TAVI approach, *n* (%)				
Transfemoral	266 (74)	77 (86)	44 (79)	0.053
Transapical and subclavian	91 (26)	12 (14)	12 (21)	0.053
Valve type, *n* (%)				
Balloon expandable	305 (85)	53 (60)∗	42 (75)	<**0**.**001**
Self-expanding	52 (15)	36 (40)∗	14 (25)	<**0**.**001**
ECG				
Sinus rhythm, *n* (%)	281 (8)	66 (74)	44 (79)	0.646
Atrial fibrillation, *n* (%)	76 (21)	23 (26)	12 (21)	
PR interval (ms)	180 ​± ​32	196 ​± ​41∗	185 ​± ​30	**0**.**002**
QRS duration (ms)	101 (92-112)	109 (98-138)∗	93 (87-104)∗^,^†	<**0**.**001**
First degree AVB, *n* (%)	60 (21)	31 (46)∗	10 (23)†	<**0**.**001**
RBBB, *n* (%)	28 (8)	23 (26)	-	<**0**.**001**
IVCD, *n* (%)	102 (29)	42 (47)∗	6 (11)∗^,^†	<**0**.**001**
Echocardiography				
AV peak velocity (m/s)	4.0 ​± ​0.8	4.1 ​± ​0.8	3.9 ​± ​0.9	0.439
AV mean gradient (mmHg)	42 ​± ​17	43 ​± ​17	42 ​± ​21	0.831
Aortic valve area (cm^2^)	0.83 ​± ​0.30	0.82 ​± ​0.27	0.82 ​± ​0.31	0.944
LV mass index (g/m^2^)	121 ​± ​37	127 ​± ​36	113 ​± ​36	0.087
LVEDV (mL)	95 ​± ​41	94 ​± ​36	86 ​± ​37	0.295
LVESV (mL)	47 ​± ​30	46 ​± ​25	44 ​± ​31	0.742
LV EF (%)	53 ​± ​11	53 ​± ​11	52 ​± ​13	0.658
LV GLS (|%|)	14.2 ​± ​4.2	14.5 ​± ​3.7	15.1 ​± ​3.4	0.339
RV dysfunction, *n* (%)	104 (31)	25 (29)	15 (29)	0.919
Significant AR, *n* (%)	69 (19)	19 (21)	9 (16)	0.736
Significant MR, *n* (%)	64 (18)	16 (18)	5 (9)	0.236
Significant TR, *n* (%)	48 (14)	22 (25)∗	9 (16)	**0**.**029**

*Notes*. Values are mean ​± ​SD, median (interquartile range), or n (%). *p*-values <0.05 were considered statistically significant and are shown in bold.

Abbreviations: AR, aortic regurgitation; AV, aortic valve; AVB, atrioventricular block; BMI, body mass index; CA, conduction abnormality; COPD, chronic obstructive pulmonary disease; ECG, electrocardiogram; eGFR, estimated glomerular filtration rate; GLS, global longitudinal strain; IVCD, intraventricular conduction delay; LBBB, left bundle branch block; LV EF, left ventricular ejection fraction; LV, left ventricle; LVEDV, left ventricular end-diastolic volume; LVESV, left ventricular end-systolic volume; MI, myocardial infarction; MR, mitral regurgitation; NYHA, New York Heart Association; PAD, peripheral artery disease; PPM, permanent pacemaker; RBBB, right bundle branch block; RV, right ventricular; TAVI, transcatheter aortic valve implantation; TR, tricuspid regurgitation.

∗ *p* ​< 0.05 on Bonferroni correction vs. no-CA group.

† *p* ​< ​0.05 on Bonferroni correction vs. new PPM group.

For the preprocedural ECG, the initial rhythm was more often sinus (79%), without significant difference between the groups. The PPM group had a longer PR interval and consequently first-degree AV block, a longer QRS duration, and more frequent IVCD vs. patients in the no-CA group. As for echocardiographic characteristics, AS severity, LV function, and RV function did not differ between the groups. Only tricuspid regurgitation varied, as it was more frequent in the PPM group as compared to the no-CA group (25% vs. 14%, *p* = 0.029).

#### One-Year Follow-Up Characteristics

Table 2 compares the clinical and echocardiographic characteristics at 1-year follow-up among the groups. Significant symptoms (NYHA III-IV) at follow-up were more common in the PPM group and in the permanent LBBB group compared to the no-CA group (11 vs. 3%, *p* = 0.029). For ECG characteristics, more than half (60%) of patients who received a PPM within 1 year had paced rhythm. When it was possible to measure in the follow-up ECG (non-paced rhythm), the PR interval was longer for the PPM group compared to the no-CA group, and QRS duration was significantly longer for both the PPM and permanent LBBB groups compared to the no-CA group (*p* < ​0.001).

**Table 2:** One-year follow-up clinical and echocardiographic characteristics according to the subgroups of CA

Variables	No-CA	PPM	Permanent LBBB	*p*-value
	(n ​= ​357)	(n = ​89)	(n ​= ​56)	
NYHA at 1 year, *n* (%)				**0**.**039**
I-II	328 (97)	82 (94)	50 (89)	
III-IV	11 (3)	5 (6)	6 (11)∗	
ECG at 1-year				
Sinus rhythm, *n* (%)	277 (78)	29 (32)	42 (75)	<**0**.**001**
Atrial fibrillation, *n* (%)	80 (22)	7 (8)	14 (25)	
Paced rhythm, *n* (%)	-	53 (60)	-	
PR interval (ms)	186 ​± ​32	209 ​± ​50∗	199 ​± ​29	<**0**.**001**
QRS duration (ms)	105 (96-116)	131 (107-156)∗	147 (138-156)∗	<**0**.**001**
First degree AVB, *n* (%)	78 (28)	14 (48)	19 (45)	**0**.**012**
Echocardiography at 1-year				
AV mean gradient (mmHg)	10 ​± ​5	10 ​± ​6	10 ​± ​4	0.742
LV mass index (g/m^2^)	96 ​± ​26	100 ​± ​24	96 ​± ​28	0.554
LV EDV (mL)	79 ​± ​30	86 ​± ​30	88 ​± ​30	**0**.**025**
LV ESV (mL)	34 ​± ​19	41 ​± ​19∗	42 ​± ​22∗	<**0**.**001**
LVEF (%)	58 ​± ​9	54 ​± ​8∗	54 ​± ​11∗	<**0**.**001**
LV GLS (|%|)	17.1 ​± ​3.7	15.1 ​± ​3.5∗	15.3 ​± ​4.3∗	<**0**.**001**
Significant AR, *n* (%)	47 (14)	10 (12)	7 (13)	0.872
Significant MR, *n* (%)	68 (20)	33 (38)∗	16 (29)	**0**.**001**
Significant TR, *n* (%)	64 (19)	32 (37)∗	10 (18)	**0**.**001**

*Notes*. Values are mean ​± ​SD, median (interquartile range), or n (%). *p*-values <0.05 were considered statistically significant and are shown in bold.

Abbreviations: AR, aortic regurgitation; AV, aortic valve; AVB, atrioventricular block; CA, conduction abnormality; ECG, electrocardiogram; GLS, global longitudinal strain; LBBB, left bundle branch block; LV EF, left ventricular ejection fraction; LV, left ventricle; LVEDV, left ventricular end-diastolic volume; LVESV, left ventricular end-systolic volume; MR, mitral regurgitation; NYHA, New York Heart Association; PPM, permanent pacemaker; TAVI, transcatheter aortic valve implantation; TR, tricuspid regurgitation.

∗ *p* < ​0.05 on Bonferroni correction vs. no-CA group.

In terms of echocardiographic findings at 1 year, left ventricular ejection fraction (LVEF) of patients with PPM and LBBB were lower compared to the no-CA group (54% and 54% vs. 58%, *p* ​< ​0.01), and GLS was likewise lower in both groups as compared to the no-CA group (15% and 15% vs. 17%, *p* ​< 0.01). For valvular regurgitation, significant mitral regurgitation and tricuspid regurgitation were more common in the PPM group (38% and 37%, respectively, *p* = 0.01).

**Figure 3:**
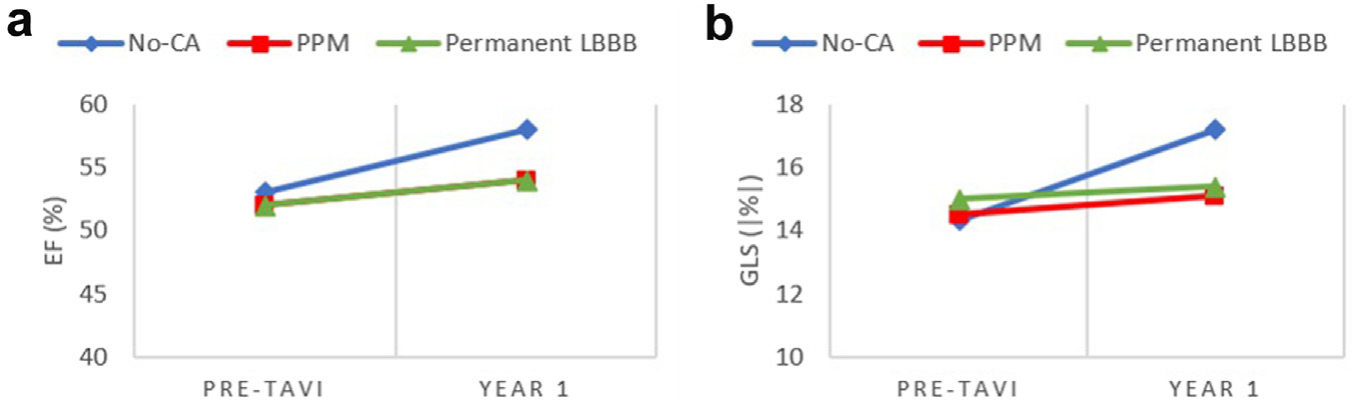
**Change in LV function from baseline to 1**-**year follow**-**up**. Graphs illustrate the change in (a) LV ejection fraction and (b) global longitudinal strain from baseline to 1-year follow-up according to the subgroups. Abbreviations: CA, conduction abnormality; EF, ejection fraction; GLS, global longitudinal strain; LBBB, left bundle branch block; LV, left ventricle; PPM, permanent pacemaker; TAVI, transcatheter aortic valve implantation.

When comparing the changes in LV echocardiographic parameters pre-TAVI and at 1-year follow-up, LVMI, LVEF, and GLS all significantly improved at follow-up (Table 3). However, when stratifying according to the CA groups, several differences were depicted: although the decrease in LV mass was similar across the subgroups, the change (improvement) in LVEF and GLS was significant only for the no-CA group and not for the PPM and permanent LBBB groups (Figure 3).

**Table 3:** Changes in LV echocardiographic parameters over time for the overall population and stratified according to the 3 subgroups

	Pre-TAVI	1-year follow-up	*p*-value
LV mass index (g/m^2^)			
Overall population	121 ​± ​37	97 ​± ​26	<**0**.**001**
No-CA	121 ​± ​37	97 ​± ​26	<**0**.**001**
PPM	128 ​± ​36	99 ​± ​24	<**0**.**001**
Permanent LBBB	113 ​± ​36	96 ​± ​28	<**0**.**001**
LV ejection fraction (%)			
Overall population	52 ​± ​11	57 ​± ​9	<**0**.**001**
No-CA	53 ​± ​11	58 ​± ​9	<**0**.**001**
PPM	52 ​± ​11	54 ​± ​8	0.072
Permanent LBBB	52 ​± ​13	54 ​± ​11	0.171
LV GLS (|%|)			
Overall population	14.4 ​± ​4.0	16.6 ​± ​3.9	<**0**.**001**
No-CA	14.3 ​± ​4.1	17.2 ​± ​3.7	<**0**.**001**
PPM	14.5 ​± ​3.7	15.1 ​± ​3.5	0.110
Permanent LBBB	15.0 ​± ​3.4	15.4 ​± ​4.4	0.483

*p*-values <0.05 were considered statistically significant and are shown in bold.

Abbreviations: CA, conduction abnormality; GLS, global longitudinal strain; LBBB, left bundle branch block; LV, left ventricle; LVEF, left ventricular ejection fraction; PPM, permanent pacemaker; TAVI, transcatheter aortic valve implantation.

#### Association of CA and Outcome

**Figure 4:**
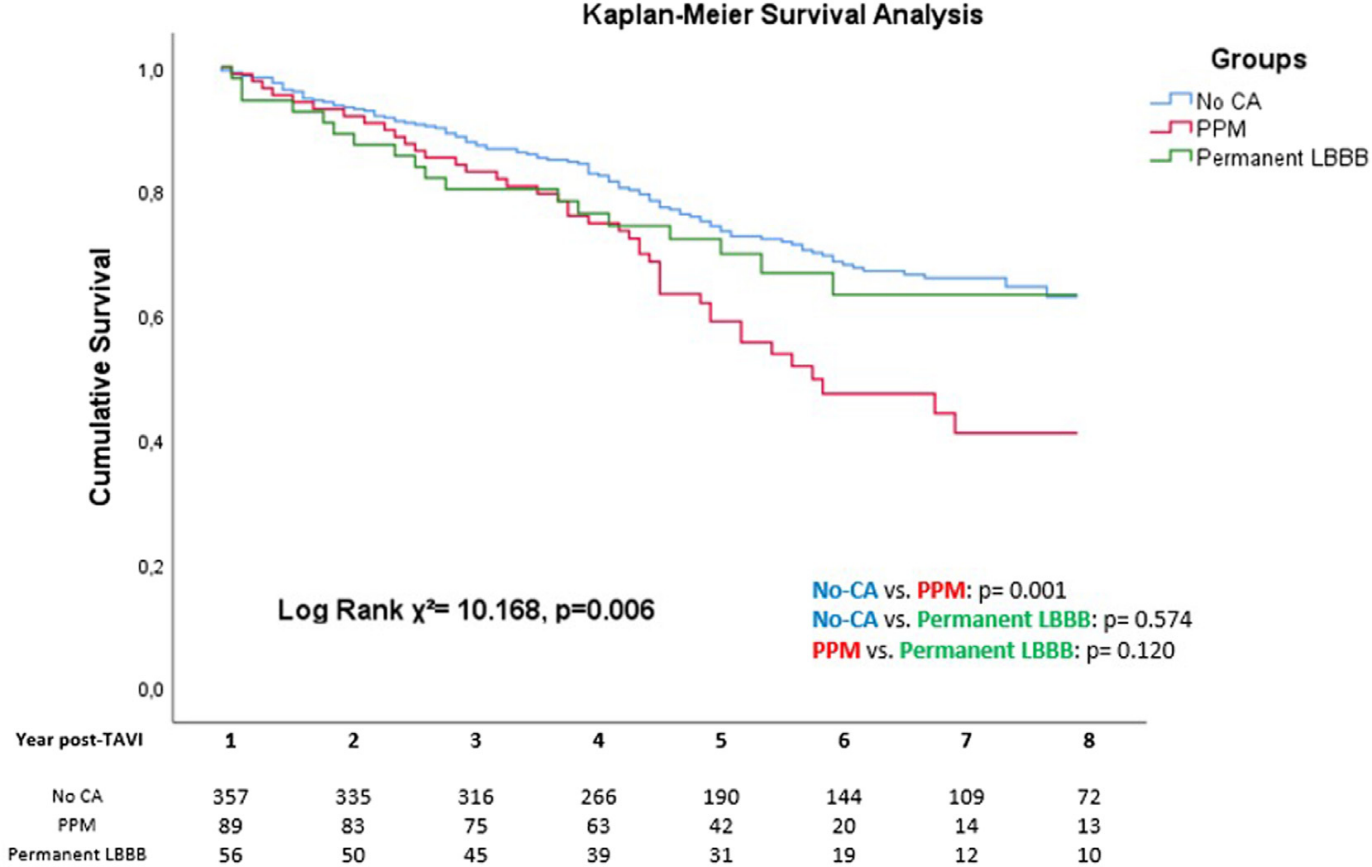
**Long**-**term outcomes of CA post**-**TAVI**. Kaplan-Meier survival curves starting at 1-year follow-up after TAVI showing a difference in all-cause mortality over time in the 3 CA groups used in the study. Abbreviations: CA, conduction abnormality; LBBB, left bundle branch block; PPM, permanent pacemaker; TAVI, transcatheter aortic valve implantation.

During a median follow-up of 49 months from the first-year follow-up after TAVI (interquartile range: 34-74 months), a total of 166 events were recorded. The Kaplan-Meier curves showed a significant difference in all-cause mortality (ꭓ2 ​= ​10.168, *p* = 0.006), specifically between patients who received a PPM as compared to those without CA (*p* = 0.001, Figure 4).

The univariable Cox regression analysis is presented in Table 4. Among the 3 subgroups, the PPM group was significantly associated with worse outcome as compared to the no-CA group. Other factors with significant hazard ratios were baseline EuroSCORE II, baseline QRS duration, and right bundle branch block or IVCD. Of the 1-year follow-up variables, NYHA class, QRS duration, and some echocardiographic parameters including GLS were also significantly associated with the outcome. Significant variables at the univariate analysis were included in the multivariable analysis (Table 4), but parameters at 1-year follow-up were prioritized (see also Statistical Methods). The analysis showed that the PPM group (hazard ratio [HR]: 1.626, *p* = 0.015), baseline EuroSCORE II (HR: 1.073, *p* = 0.002), worse NYHA class at 1 year (HR: 4.461, *p* ​< ​0.001), and LV GLS at 1 year (HR: 0.951, *p* = 0.034) were independently associated with all-cause mortality.

**Table 4:** Univariable and multivariable Cox proportional hazard model for prediction of all-cause mortality

	Univariate analysis		Multivariable analysis	
	Hazard ratio (95% CI)	*p*-value	Hazard ratio (95% CI)	*p*-value
Age (y)	0.986 (0.966-1.007)	0.183		
Gender	1.334 (0.980-1.816)	0.067		
BMI (kg/m^2^)	1.004 (0.970-1.038)	0.829		
EuroSCORE II	1.083 (1.043-1.125)	<**0**.**001**	1.073 (1.026-1.121)	**0**.**002**
TAVI valve type				
Balloon expandable	1.482 (0.961-2.285)	0.075		
Self-expanding	0.675 (0.438-1.041)	0.075		
Baseline ECG				
First degree AVB	1.282 (0.871-1.886)	0.207		
RBBB	1.907 (1.262-2.881)	**0**.**002**		
IVCD	1.685 (1.236-2.298)	**0**.**001**		
PR interval	1.003 (0.998-1.008)	0.230		
QRS duration	1.011 (1.005-1.017)	<**0**.**001**		
Baseline echocardiography				
AV mean gradient	0.997 (0.988-1.006)	0.484		
Aortic valve area (cm^2^)	0.905 (0.542-1.511)	0.703		
LV mass index	1.000 (0.996-1.004)	0.999		
LVEF	0.993 (0.981-1.006)	0.316		
LV GLS	0.984 (0.947-1.022)	0.407		
CA subgroup at 1 y				
No-CA	Reference	**0**.**007**	Reference	**0**.**040**
PPM	1.775 (1.240-2.539)	**0**.**002**	1.626 (1.098-2.409)	**0**.**015**
Permanent LBBB	1.155 (0.701-1.904)	0.572	0.991 (0.584-1.680)	0.973
At 1 y				
NYHA III-IV	4.210 (2.501-7.086)	<**0**.**001**	4.461 (2.610-7.626)	<**0**.**001**
QRS duration	1.010 (1.004-1.017)	**0**.**002**		
Echocardiography at 1 y				
AV mean gradient	1022 (0.994-1.051)	0.126		
LV mass index	1.008 (1.002-1.013)	**0**.**009**	1.000 (0.993-1.007)	0.957
LVEDV	1.007 (1.002-1.011)	**0**.**008**		
LVESV	1.009 (1.002-1.016)	**0**.**013**		
LVEF	0.987 (0.971-1.003)	0.103		
LV GLS	0.933 (0.897-0.971)	<**0**.**001**	0.951 (0.907-0.996)	**0**.**034**
Significant AR	1.378 (0.922-2.059)	0.118		
Significant MR	1.183 (0.837-1.672)	0.341		
Significant TR	1.053 (0.727-1.525)	0.784		
Pacing percentage at 1 y				
<0%	Reference	0.259		
1%-40%	2.081 (0.745-5.812)	0.162		
>40%	2.262 (0.847-6.045)	0.104		

*p*-Values <0.05 were considered statistically significant and are shown in bold.

Abbreviations: AR, aortic regurgitation; AV, aortic valve; AVB, atrioventricular block; BMI, body mass index; CA, conduction abnormality; ECG, electrocardiogram; GLS, global longitudinal strain; IVCD, intraventricular conduction delay; LV, left ventricle; LVEDV, left ventricular end-diastolic volume; LVEF, left ventricular ejection fraction; LVESV, left ventricular end-systolic volume; MR, mitral regurgitation; NYHA, New York Heart Association; PPM, permanent pacemaker; RBBB, right bundle branch block; RV, right ventricle; TAVI, transcatheter aortic valve implantation; TR, tricuspid regurgitation.

## Discussion

The main findings of this study can be summarized as follows: 1) New CA after TAVI are dynamic within the first year after intervention, with a notable decrease in the prevalence of LBBB; 2) LV function, as assessed by both EF and GLS, improves 1 ​year after TAVI, but the change is significant only in patients with no-CA group; and 3) PPM implantation, but not permanent LBBB, is independently associated with worse outcome beyond the first year, together with increased EuroSCORE II, more symptoms, and reduced LV GLS.

### CA Changes Over Time After TAVI

Conduction disturbances remain a relatively frequent complication after TAVI. The rates of PPM implantation vary according to different studies from 3% to 36%, while new-onset LBBB varies from 4% to 65%.^4^ These rates, however, reflect only patients who experience CA immediately postprocedure or within the first 48 ​hours. Despite the awareness that in a significant number of patients, CA can improve over time with the resolution of the inflammation,^4^ most studies focused on the impact of CA developed acutely after TAVI regardless of their persistence. A different approach is applied in the current study by assessing how these CA change within the first year and evaluating how the persistence of these disturbances relates to outcomes. In line with previous studies,^4^ PPM implantation at discharge was 12% and new-onset LBBB was 17%. Interestingly, at the 1-year follow-up, there were 18% with PPM, while only 11% with permanent LBBB.

The CA dynamic may be explained by mechanical manipulation during the AV procedure causing inflammation, edema, or ischemia of the His bundle or the left bundle branch, which traverse close to the aortic annulus.^4^ If and when these local damages resolve is unpredictable, and therefore, the onset and/or resolution of CA are variable as well.

When patients develop LBBB after TAVI, 85% to 94% of these LBBB occur already periprocedurally, but only 44% to 65% persist until discharge or 30 days.^11^ The prevalence of LBBB after 30 days has not been evaluated extensively, and little is known about its impact on long-term outcomes. The current study showed that a third of patients demonstrate LBBB recovery, with 37% labeled initially as persistent LBBB moving to the no-CA group at 1 year. This is in agreement with the findings of Faroux et al., who noted a 33% recovery of LBBB at 1 year but in a small patient cohort (n = 153) with new-onset LBBB^12^; however, the authors did not report the impact of LBBB recovery on outcomes. LBBB can also progress toward HAVB and eventually require a PPM in 5% to 14% of patients.^11^ For the current study’s cohort, 7% of those with LBBB postprocedure received a pacemaker before discharge, while 2% of those discharged with LBBB received a PPM within 1 ​year. Given this low likelihood for patients with LBBB to need a PPM, guidelines recommend electrophysiologic testing or long-term monitoring instead of immediate PPM implantation.^13^

Similar to LBBB, HAVB occurs primarily periprocedurally, which in 60% to 96% of patients is within the first 24 ​hours, and 85% to 90% receive a PPM within 7 days.^11^ The course after that is likewise seldom assessed. In the study by De-Torres-Alba et al., 22% (n = 17) of CA requiring PPM implantation occurred after 48 ​hours, and 10% (n = 8) after 5 days; among the patients who developed CA requiring PPM after 48 ​hours, 24% had no prior CA.^14^ However, this study limited the observation to the first week postprocedure. Our study in turn documented that 21% received a PPM after the first week from TAVI—a sizable number that should still be studied when examining long-term outcomes.

The current study was also able to demonstrate that pacing percentage tended to decrease over time, in concordance with previous studies. In our cohort, 45% of patients had >40% pacing at 1 year, as compared to 54% at discharge. A literature review by Ravaux et al. determined that up to 50% of patients with PPM did not show PPM dependency at 1 year,^15^ while Costa et al.^16^ showed an even smaller percentage of 33%. This decrease in PPM dependency, along with the unclear association of PPM with mortality, has led to stricter recommendations in PPM implantation post-TAVI.^13^

### Impact of CA on LV Function and Long-Term Outcomes

The dynamicity of these CA over time and the different definitions of LBBB and PPM implantation post-TAVI have created conflicting evidence on their impact on mortality. Data are particularly scarce regarding long-term follow-up of patients with CA (especially LBBB), since most studies limited the analysis to 1 or 2 years after TAVI. Among the studies with longer follow-up time, Costa et al. demonstrated in patients with PPM an increase in all-cause mortality at 6 years,^16^ while Chamandi et al. did not show an effect of PPM on mortality for a follow-up of 4 years^17^; still, both studies included patients who received PPM only within 30 days. Regarding the impact of LBBB on long-term outcomes, similar conflicting results are reported. Chamandi et al. did not find a significant association between new-onset LBBB at discharge with mortality at 3 years of follow-up,^18^ while Houthuizen et al. noted a higher all-cause mortality in patients who developed LBBB within 7 days post-TAVI within a follow-up of 450 days.^19^

The current study tried to clarify this aspect by including a large patient population and defining CA at a landmark point of 1 ​year after TAVI. Similar findings to the study of Costa et al.^16^ were observed as patients who received a PPM were found to have a higher mortality risk compared to patients with no-CA or permanent LBBB, within a follow-up of 4 years (therefore 5 years after TAVI). Previous studies have already suggested that the association between PPM and mortality occurs through cardiac dyssynchrony secondary to RV pacing, which causes a reduction in cardiac output and myocardial perfusion, including sympathetic activation and endothelial dysfunction, referred to as “pacing-induced heart disease.”^20^ Cardiac dyssynchrony also hampers postprocedural normalization of cardiac function and may even induce further decline.^20^ However, it is also possible that the association between PPM and mortality may have other causes, such as the ischemic injury to the conduction system itself, rather than being induced by pacing.^20^

In addition, the current study showed that permanent LBBB post-TAVI was not significantly associated with mortality, which is in concordance with the findings of Chamandi et al.^18^ Unlike most studies that focused on the presence of LBBB within the first week, we assessed the effect of LBBB persistence at 1 ​year on long-term outcomes. Other studies that identified an association between LBBB and mortality explained this relationship by possible progression to HAVB or by cardiac dyssynchrony^19^—mechanisms that may have resolved at 1 ​year.

Finally, this study evaluated the impact of CA post-TAVI on LV function in the first year after intervention. Dolci et al. demonstrated that EF improved after TAVI for the overall population, but significantly only in patients without CA.^21^ However, follow-up duration was restricted to 6 months, and only EF was used to assess LV function. Similarly, but by using both EF and GLS, the current study showed that both parameters improved for the entire population 1 year after TAVI. However, the improvement was significant only for patients without CA. These results may partially explain the difference in outcomes among the subgroups, especially since a significant association was demonstrated between GLS at 1 year and mortality.

The findings of the current study support and expand existing knowledge on the significant impact of CA, particularly when leading to PPM implantation, on long-term outcome after TAVI. Monitoring CA up to 1 ​year after the procedure becomes, therefore, important, as CA may change as compared to immediately post-TAVI. In addition, when indications for PPM implantation present, these patients may require closer follow-up, including LV function assessment and particularly using GLS as the most reflective of subclinical changes and independently associated with mortality. These findings also support current efforts to improve devices and implantation techniques in order to prevent the development of CA after TAVI and to minimize the impact of pacing in patients who still develop these CA. These observations are particularly important to be taken into account when considering TAVI in low-risk or asymptomatic patients.

### Study Limitations

As a retrospective single-center study, there are limitations imposed by the study design, particularly on generalizability of results. Larger prospective studies are needed to confirm these findings and establish more definitive causalities. Second, in order to ensure a large number of included patients and sufficient long-term follow-up, the inclusion period was long and relatively dated (2007 to 2019) and may therefore represent a limitation in terms of applicability of the results to the new generation of TAVI prosthesis. Also, guidelines for TAVI indication, procedure specifications, and PPM implantation have changed and could have an impact on patient selection and outcomes. Third, the study investigated outcomes beyond the first year, which caused 292 patients to be excluded at the landmark point of 1 year, with a potential selection bias. However, as presented in the Supplemental File, no substantial differences were observed between patients included and excluded in the long-term analyses. Lastly, missing data could not be completely avoided and could have had some effect on the results.

## Conclusion

After TAVI, CAs remain relatively frequent but with a dynamic nature, which necessitates regular ECG follow-up, especially within the first year. PPM implantation is associated with less or no improvement in LV function after TAVI and increased risk for long-term mortality, suggesting the need for a closer follow-up of these patients, potentially using GLS to monitor LV function.

## Ethics Statement

This research was performed in accordance to relevant research ethical guidelines and received approval from the Leiden University Medical Center Institutional Review Board as a retrospective study.

## Funding

This research received a study grant from 10.13039/100006520Edwards Lifesciences (Irvine, California).

## Disclosure Statement

The Department of Cardiology of Leiden University Medical Center received research grants from Abbott Vascular, Alnylam, Bayer, Biotronik, Bioventrix, Boston Scientific, Edwards Lifesciences, GE Healthcare, Medtronic, Pie Medical, Medis, Pfizer, and Novartis. A. P. Chua, R. Myagmardorj, and T. Nabeta received research grants from Turku PET Centre. N. Ajmone Marsan received speaker fees from Abbott Vascular, Philips Ultrasound, Omron, Pfizer, and GE Healthcare. J. J. Bax received speaker fees from Abbott Vascular, Edwards Lifesciences, and Omron. The other authors had no conflicts to declare.
