# Supplementary material for: Conduction Disorders After Transcatheter Aortic Valve Implantation: Evolution Over Time and Association With Long-Term Outcomes
Source: Struct Heart. 2025 Feb 17;9(5):100428. doi: 10.1016/j.shj.2025.100428 (PMC12168359; doi:10.1016/j.shj.2025.100428)
Supplement: Supplementary Appendix [file mmc1.docx]

Conduction disorders after transcatheter aortic valve implantation: evolution over time and association with long-term outcomes

**Supplementary Material**

*Supplementary Table 1*………………………………………………………………………...2

**Supplemental Table 1:** Comparison of baseline characteristics of patients included in the current study versus the patients that were excluded due to death or insufficient follow-up.

|  | Study Population  (n= 502) | Excluded Patients  (n= 292) | P-value |
| --- | --- | --- | --- |
| Age (years) | 80 ± 7 | 80 ± 8 | 0.969 |
| Male sex, *n* (%) | 265 (53) | 152 (52) | 0.842 |
| BMI (kg/m2) | 26.7 ± 4.4 | 26.2 ± 4.8 | 0.143 |
| EuroSCORE II (%) | 3.03 (1.93- 5.00) | 2.57 (1.59- 3.92) | **<0.001** |
| NYHA, *n*(%) |  |  | 0.054 |
| I-II | 203 (40) | 135 (48) |  |
| III-IV | 299 (60) | 149 (53) |  |
| Co-morbidities |  |  |  |
| Hypertension, *n* (%) | 381 (76) | 217 (75) | 0.676 |
| Dyslipidemia, *n* (%) | 328 (65) | 169 (58) | **0.042** |
| Diabetes Mellitus, *n* (%) | 136 (27) | 84 (29) | 0.591 |
| Coronary artery disease, *n* (%) | 296 (59) | 147 (51) | **0.021** |
| Previous MI, *n* (%) | 98 (20) | 52 (18) | 0.567 |
| Stroke, *n* (%) | 86 (22) | 42 (18) | 0.256 |
| PAD, *n* (%) | 144 (29) | 77 (27) | 0.596 |
| Atrial Fibrillation, *n* (%) | 132 (26) | 85 (29) | 0.375 |
| Smoking, *n* (%) | 105 (21) | 72 (25) | 0.203 |
| eGFR (ml/min/1.73 m^2^) | 65 ± 22 | 60 ± 23 | **0.007** |
| PROCEDURAL VARIABLES | | | |
| TAVI Approach, *n* (%) |  |  |  |
| Transfemoral | 387 (77) | 212 (73) | 0.156 |
| Transapical & Subclavian | 115 (23) | 80 (27) | 0.156 |
| Valve Type, *n* (%) |  |  |  |
| Balloon expandable | 400 (80) | 238 (82) | 0.360 |
| Self- expanding | 102 (20) | 51 (18) | 0.360 |
| BASELINE ECG | | | |
| Sinus rhythm, *n* (%) | 391 (78) | 215 (74) | 0.173 |
| Atrial fibrillation, *n* (%) | 111 (22) | 77 (26) |  |
| PR interval (ms) | 183 ± 34 | 180 ± 33 | 0.380 |
| QRS duration (ms) | 101 (91-113) | 102 (91-117) | 0.177 |
| First degree AVB, *n* (%) | 101 (26) | 53 (25) | 0.726 |
| RBBB, *n* (%) | 51 (10) | 36 (12) | 0.345 |
| IVCD, *n* (%) | 150 (30) | 87 (30) | 0.980 |
| BASELINE ECHOCARDIOGRAPHY | | | |
| AV peak velocity (m/s) | 4.0 ± 0.8 | 4.0 ± 0.7 | 0.979 |
| AV mean gradient (mmHg) | 42 ± 18 | 42 ± 16 | 0.972 |
| Aortic valve area (cm^2^) | 0.83 ± 0.29 | 0.82 ± 0.29 | 0.878 |
| LV mass index (g/m^2^) | 121 ± 37 | 125 ± 35 | 0.132 |
| LVEDV (ml) | 94 ± 39 | 105 ± 46 | **<0.001** |
| LVESV (ml) | 47 ± 29 | 53 ± 36 | **0.018** |
| LV EF (%) | 53 ± 11 | 52 ± 12 | 0.719 |
| LV GLS (\|%\|) | 14.4 ± 4.0 | 13.4 ± 3.8 | **0.003** |
| RV dysfunction , *n* (%) | 144 (30) | 83 (32) | 0.521 |
| Significant AR, *n* (%) | 97 (19) | 63 (24) | 0.131 |
| Significant MR, *n* (%) | 85 (17) | 52 (20) | 0.361 |
| Significant TR, *n* (%) | 79 (16) | 43 (17) | 0.846 |

Values are mean ± SD, median (IQR), or n(%). P-values <0.05 were considered statistically significant and are shown in bold.

AR, Aortic Regurgitation; AV, Aortic Valve; AVB, Atrioventricular Block; BMI, Body Mass Index; CA, conduction abnormality; ECG, Electrocardiogram; eGFR, Estimated Glomerular Filtration Rate; GLS, Global Longitudinal Strain; IVCD, Intraventricular Conduction Delay; LV, Left Ventricle; LVEDV, Left Ventricular End Diastolic Volume; LV EF- Left ventricular ejection fraction; LVESV, Left Ventricular End Systolic Volume; MI, Myocardial Infarction; MR, Mitral Regurgitation; NYHA, New York Heart Association Heart Failure Classification; PAD, Peripheral Artery Disease; RBBB, Right bundle branch block; RV, Right Ventricle; TAVI, Transcatheter Aortic Valve Implantation; TR, Tricuspid Regurgitation.
